# From dogma to data: charting a path forward for clinico-immunological research in Ethiopian cutaneous leishmaniasis

**DOI:** 10.1186/s40249-025-01398-2

**Published:** 2026-01-16

**Authors:** Thao-Thy Pham, Saskia van Henten, Mikias Woldetensay, Mezgebu Silamsaw Asres, Eleni Ayele, Paul M. Kaye, Malgorzata Anna Domagalska, Jean-Claude Dujardin, Johan van Griensven, Wim Adriaensen

**Affiliations:** 1 Department of Clinical Sciences, Institute of Tropical Medicine Antwerp, Antwerp, Belgium; 2 Department of Dermatology and Venereology, University of Gondar, Gondar, Ethiopia; 3 Department of Internal Medicine, University of Gondar, Gondar, Ethiopia; 4 Leishmania Research and Treatment Center, University of Gondar, Gondar, Ethiopia; 5 York Biomedical Research Institute and Hull York Medical School, University of York, York, UK; 6 Department of Biomedical Sciences, Institute of Tropical Medicine Antwerp, Antwerp, Belgium

**Keywords:** Cutaneous leishmaniasis, *Leishmania aethiopica*, Immunopathogenesis, Leishmaniasis, Clinico-immunology, Immunoparasitology, Ethiopia

## Abstract

**Background:**

Rooted in long-standing assumptions and adapted from classifications mainly used for Latin American cutaneous leishmaniasis (CL), the nationally recommended clinical categories in Ethiopia for CL remain limited to localized cutaneous leishmaniasis (LCL), mucocutaneous leishmaniasis (MCL), and diffuse cutaneous leishmaniasis (DCL). However, these categories are associated with immune mechanisms which have not been validated in the Ethiopian context and thus risk misrepresenting the true clinical and immunopathological diversity. In this opinion piece, we will therefore outline key knowledge gaps and challenges in current clinico-immunological research on Ethiopian CL.

**Main body:**

In Ethiopia, *Leishmania aethiopica* is often assumed as the causative agent of these ‘LCL’, ‘MCL’ and ‘DCL’ forms, yet significant gaps in knowledge urge caution. For example, adoption of this ‘LCL’, ‘MCL’ and ‘DCL’ terminology and the associated immune mechanisms has led to inconsistent results. Most immunological studies on Ethiopian CL have focused on peripheral blood, resulting in little information about immune processes in the lesion, the original site of infection. Adding to the complexity, other species (including *L. major*, *L. donovani* and *L. tropica* as well as *L. aethiopica* hybrids) also circulate, with reports of *Leishmania* RNA virus co-infection. To address these challenges, we propose a multidimensional approach that combines standardized clinical documentation with appropriate lesion and blood sampling for in-depth profiling of both immune responses and parasite diversity. Adopting this holistic approach will require inclusive (inter)national collaborations with a focus on promoting equitable biobank and data sharing which will strengthen local research capacities.

**Conclusions:**

By addressing the main challenges and knowledge gaps in current clinico-immunological research through a multidimensional approach, this opinion aims to provide the tools to achieve a better and unbiased understanding of the immunopathogenesis of Ethiopian CL, a severe yet under-investigated disease. Such progress is essential for improving CL management in Ethiopia and aligns with the World Health Organization priority on controlling CL.

**Graphical Abstract:**

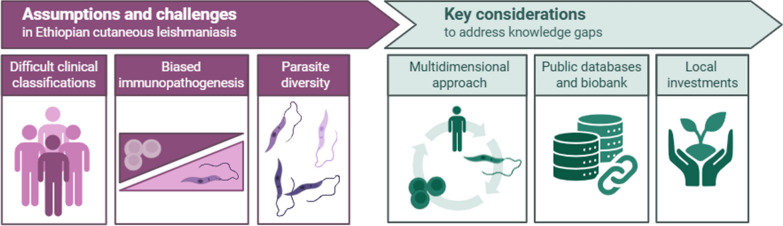

## Background

Ethiopia has one of the highest incidences of cutaneous leishmaniasis (CL) in East Africa with up to 40,000 new cases each year [1]. In the Ethiopian highlands, an expansive plateau dominates the landscape where zoonotic transmission of *Leishmania* typically occurs through the sand fly vectors *Phlebotomus pedifer* and *Phlebotomus longipes*, with hyraxes reported to act as the main reservoirs [2]. Individuals in these endemic areas live in poor socio-economic conditions, and frequent outdoor exposure due to farming, herding, and/or cultural gatherings contributes to their heightened risk of infection. Although sporadic reports of *Leishmania major, L. donovani* and *L. tropica* have been described, the main infecting species for Ethiopian CL remains *L. aethiopica* [2]*.* CL caused by *L. aethiopica* presents a wide spectrum of clinical manifestations and often responds poorly to first-line treatment regimens [2]. While the clinical presentations are extremely diverse, the national treatment guidelines in Ethiopia describe only three clinical classifications: localized CL (LCL), mucocutaneous leishmaniasis (MCL), and diffuse CL (DCL)[3]. The nomenclature of these CL subcategories is well established in the *Leishmania* field, and in the context of Latin America, each category has been associated with distinct underlying immunopathological mechanisms [4].

Despite early research interest in Ethiopian CL, a recent scoping review indicated that limited research has been done on the immune responses underlying Ethiopian CL, identifying a significant knowledge gap [5–7]. Several factors have likely contributed to this gap, many of which reflect broader challenges within Ethiopia’s research infrastructure; During much of the twentieth century, the limited governmental resources were primarily directed to basic services, healthcare and agriculture, leaving little room for investment in scientific research. Research in Ethiopia has therefore largely relied on international funding, either through competitive research grants or capacity strengthening initiatives. However, recurrent political unrest in northern Ethiopia, from the Eritrean-Ethiopian war (1998–2000) and its aftermath, to the more recent Tigray civil war and its related conflicts (2020–present), has not only impaired local north-Ethiopian research infrastructure (e.g., raided lab facilities, closed transport routes and disrupted utility networks), but also flagged Ethiopia as a high-risk country in several international funding schemes [e.g., National Institutes of Health (NIH)]. These challenges, together with the absence of experimental rodent models for *L. aethiopica* infection, have resulted in researchers extrapolating existing frameworks in an attempt to bridge the knowledge gap on the immunopathogenesis in Ethiopian CL. A prominent example is the T helper 1 response (Th1)-based paradigm, which links self-healing CL, mucosal leishmaniasis and DCL to protective, excessive, and anergic Th1 responses, respectively [4]. However, this paradigm originates from mainly Latin American CL studies including other *Leishmania* species, and placing these findings into the Ethiopian context has led to ill-fitting clinical classifications with assumptions about underlying immunopathogenesis mechanisms that remain unverified.

We argue that the continued extrapolation of these assumptions on clinical classifications, immunopathogenic mechanisms and infecting parasite species, have introduced bias into Ethiopian CL research over time. To date, the immunopathogenesis driving the different clinical presentations of Ethiopian CL remains unknown. Addressing this knowledge gap is crucial for improving disease management and will guide the development of more effective treatment strategies for Ethiopian CL, which will contribute to the World Health Organization (WHO) strategic framework for CL control by 2030 [8]. In this opinion, we challenge the current established views on Ethiopian CL, expose specific knowledge gaps, and provide essential tools to perform clinico-immunology research in an unbiased, inclusive and comprehensive manner.

## Current assumptions in Ethiopian CL and their challenges

### Heterogeneity in clinical presentations

**Fig. 1:**
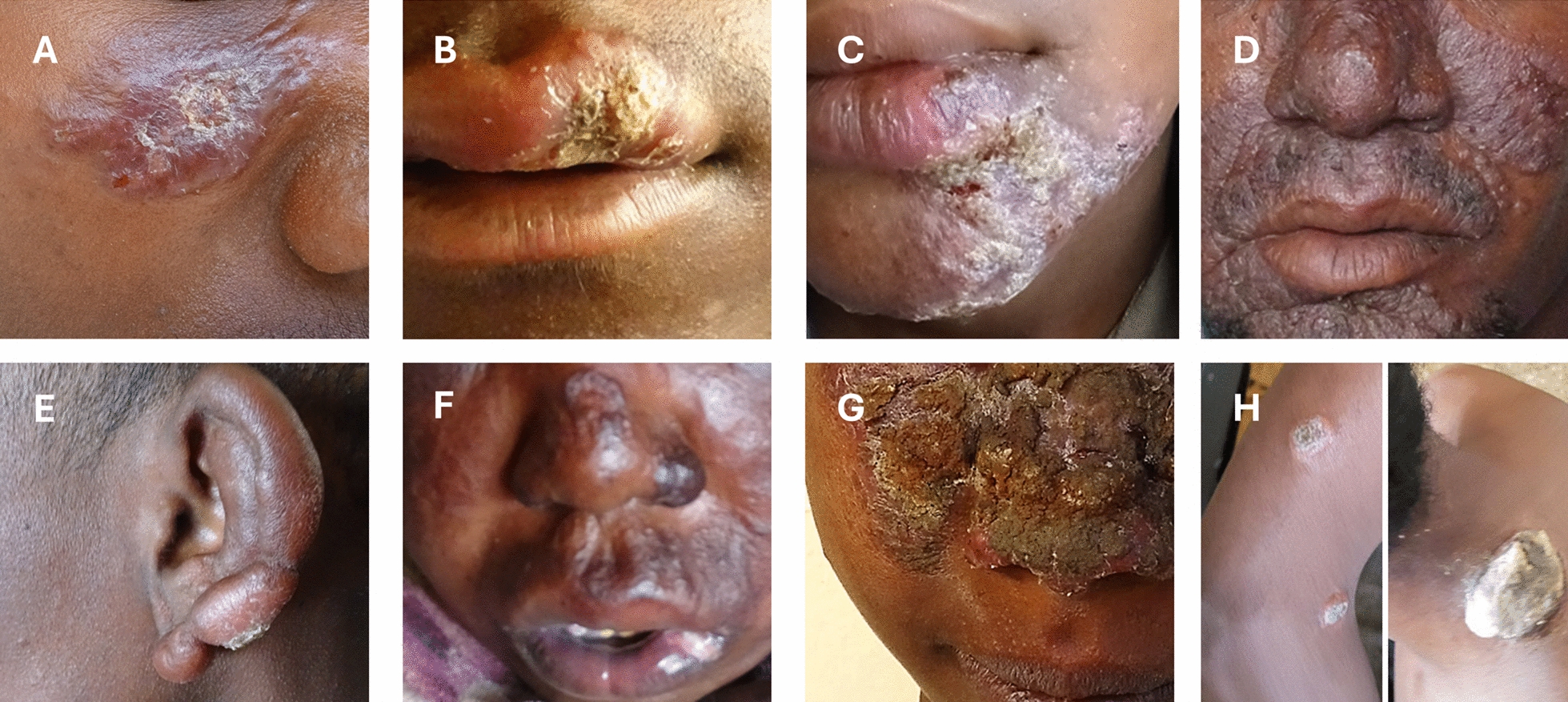
The diverse clinical manifestations of Ethiopian cutaneous leishmaniasis (CL). Example of Ethiopian cutaneous leishmaniasis (CL) patients with **A** a large solitary erythematous plaque lesion with overlying scales on right cheek, **B** a crusted singular dry ulcer over a swollen upper lip, **C** multiple facial erythematous crusted plaques along with a swollen lower lip, **D** multiple confluent plaques with satellite papules spread over the face, **E** nodular infiltration of the ear lobe (reminiscent of lepromatous leprosy), **F** diffuse facial swelling with ill-defined plaques and an ulcer over the lip, **G** large crusted lesion with subcutaneous swelling over the midfacial region, and **H** multiple well-circumscribed plaques over the right arm with a crusted plaque in the neck. All patients were enrolled within the Spatial CL study [11].

The clinical presentations of Ethiopian CL are extremely diverse (Fig. 1), yet detailed descriptions of these presentations remain scarce in the literature. Currently, official Ethiopian treatment guidelines limit these clinical presentations to three categories: (i) ‘LCL’ when the location of the (often) self-healing lesion coincides with the sand fly bite, (ii) ‘MCL’ when there is involvement of the mucosa either through a direct bite on mucosal areas or through progression from the skin to the mucosa without spontaneous healing, and (iii) ‘DCL’ when there is a chronic disease with multiple papular, nodular or plaque lesions spread over large areas of the body [9]. A limitation of this classification description is its reliance on the date and location of the sand fly bite, details that are frequently unknown to patients and therefore not requested by the physicians. Regarding treatment, localized approaches such as cryotherapy or intralesional injections with pentavalent antimonial drugs are typically used for localized small lesions (≤ 4 cm in diameter) characteristically seen in ‘LCL’ patients. In contrast, ‘MCL’ and ‘DCL’ cases as well as ‘LCL’ with lesions that are larger, multiple or in unsuitable anatomical regions for local treatment, are generally managed with systemic treatment strategies [10].

A recent survey amongst Ethiopian dermatologists observed that the ‘LCL’, ‘MCL’, and ‘DCL’ terms were most commonly used to classify patients, but not uniformly applied [12]. Problematically, ‘mucocutaneous’ versus ‘mucosal’ leishmaniasis, and ‘diffuse’ versus ‘disseminated’ were either interchangeably used or considered as separate subtypes. Other categories such as *L. recidivans* were also mentioned. Classification difficulties were largest for ‘MCL’ and ‘DCL’ patients, due to different understanding and application of these categories amongst clinicians. Within the Spatial CL study, we also encountered ambiguous cases that did not fit neatly into the ‘LCL’, ‘MCL’, or ‘DCL’ categories (Fig. 1). In the *Leishmania* field, such a spectrum of clinical manifestations is sometimes referred to as ‘tegumentary CL’. The definition for ‘tegumentary’ denotes the involvement of the body’s external covering and therefore does not include the clinical manifestations limited to internal mucosal lesions, subcutaneous swellings nor nodules without overlaying lesions. This terminology is also not employed in current guidance reports such as the Pan American Health Organization (PAHO) guidelines for the control of leishmaniasis (published in 2024)[13].

A significant caveat is the use of ‘mucocutaneous’ versus ‘mucosal’ leishmaniasis (ML) terminology in Ethiopian CL. The WHO and PAHO guidelines describe both ‘ML’ and ‘MCL’ terms to typically be the result of (i) a hematogenous metastasis after an initial and distant skin lesion, (ii) more rarely the extension of a facial skin lesion to mucosal membranes, or (iii) the direct bite of the vector on the mucous membrane, which can progress to further destroy the oronasal cavities, upper respiratory tract and larynx [13, 14]. More importantly, the PAHO guidelines emphasize that these clinical classifications are largely restricted to *Leishmania* species circulating in Latin America. Meanwhile, according to the official Ethiopian guidelines, patients are classified as ‘MCL’ when the lesion involves the mucosa either through (i) a direct bite of the vector on mucosal areas (Fig. 1B) or (ii) progression from facial skin to mucosa without spontaneous healing (Fig. 1C)[3]. A defining difference is that, in Ethiopia, these ‘MCL’ lesions are commonly confined to the mucosal borders of the nostrils and lips, and rarely cause further extensive disfigurement towards the respiratory tract or larynx [2]. An alternative term ‘oronasal leishmaniasis’ has been proposed by the WHO report in 2010 for *L. aethiopica*-induced cutaneous lesions affecting the nostrils and/or lips [14]. However, this terminology is not practiced by local physicians, and excludes lesions involving the conjunctival mucosa.

Classifying CL patients in clinical categories is valuable to inform treatment strategies, and to extrapolate research findings between different cohorts, if used consistently. We therefore recommend moving away from the simplified classification ‘LCL’, ‘MCL’, ‘DCL’ within the Ethiopian context, and indicate the need for a better defined clinical classification or scoring system that promotes diagnostic consistency between clinicians. A recent study has subdivided the ‘LCL’ classification into lesions that were contained versus those with ill-defined edges which were considered to be spreading [15]. Meanwhile, the SHARP consortium recently found that consistency in clinical assessment could be achieved for lesion size measurements and major morphological categories (plaque, nodule, or papule), but not for secondary features such as dyspigmentation, scale, or mucosal involvement [16]. Despite applying more precise criteria for their definitions (including the number of lesions, the extent of body sites involved, and the presence or absence of mucosal involvement), the SHARP consortium still used similar terminology in their proposed classifications: ‘LCL’, ‘multi-regional LCL’, ‘MCL’, ‘ML’, and ‘DCL’ [17].

Building on these insights, we propose to discontinue the use of any of these biased classification terminologies in future clinico-immunological studies on Ethiopian CL, and instead recommend performing an unbiased and comprehensive clinical assessment, as guided by the parameters outlined in Table 1, while a validated Ethiopia-specific classification is being developed. Such data-driven classifications may further integrate additional factors, including associations with underlying immune responses (potential immunological endotypes or immunotypes) and parasite species. To generate new classifications for Ethiopian CL, established methods like the ‘nominal group technique’ may be applied. This technique was recently used to define a ‘core outcome measure instrument’ for ‘LCL’, with its key clinical parameters integrated in Table 1 [18]. To support practical implementation, an additional micro-costing approach (i.e. step-by-step costing exercise) is suggested to identify which clinical observations and sampling procedures are essential, affordable, and feasible in routine Ethiopian settings.

**Table 1:** Recommended clinical parameters to record in future clinico-immunological studies on Ethiopian cutaneous leishmaniasis (CL)

*Leishmania* infection history	Number of previous CL lesions (number, each describing estimated date start–date end)			
	Anatomical site of previous lesions (mark on pictogram)			
	Past modern antileishmanial treatments (each describing estimated date start–date end)			
	Past traditional antileishmanial treatments (each describing estimated date start–date end)			
Comorbidities	Clinically suspected/reported by patient/examination in blood, stool or tissue(s)			
	☐ Leprosy	☐ HIV infection		
	☐ Tuberculosis	☐ *Plasmodium* infection		
	☐ HBV/HCV	☐ Helminth infection		
	☐ Malnutrition	☐ Non-communicable diseases (e.g., diabetes, cancer)		
		☐ Dermatological diseases (e.g., psoriasis, acne vulgaris)		
Lesion assessment	Number of lesions			
(baseline)	Size of index and other lesions (diameter width × height, perpendicular)			
	Duration of lesions (each describing estimated date start–date end)			
	Induration (diameter width × height, perpendicular with palpability score 0–9)			
	Anatomical site of lesions (mark on pictogram)			
	Lesion morphology:			
		A) Primary morphological categories		
		☐ Nodule	☐ Plaque	
		☐ Papule	☐ Ulceration	
		B) Secondary morphological categories		
		☐ Scaling	☐ Dryness	
		☐ Crusting	☐ Exfoliation	
		C) Clinical features		
		☐ Erythema	☐ Hyperpigmentation	
		☐ Overall swelling	☐ Hypopigmentation	
		☐ Induration		
		D) Mucosal involvement		
		☐ Oral labial mucosa (lips)		
		☐ Nasal vestibular mucosa (inner nose)		
		☐ Lingual mucosa (tongue)		
		☐ Palatal mucosa		
		☐ Conjunctiva		
		E) Complications		
		☐ Phymatous changes		
		☐ Macrocheilia		
		☐ Secondary bacterial infection		
		☐ Permanent loss of tissue		
Treatment outcome	Lesion assessment (see above)			
	Re-epithelization (0–100%)			
	Scar morphology:	☐ Normotrophic	☐ Hyperpigmentation	
		☐ Atrophic	☐ Hypopigmentation	
		☐ Hypertrophic/keloid		
	Overall clinical outcome (assessed by physician and patient, 0–100%)			
Administered CL treatment	Drug (combination) details			
	Duration treatment (including extension period)			
	Number of treatment cycles			
	Administration:	☐ intralesional		
		☐ intramuscular		
		☐ intravenous		
		☐ oral		

*CL* cutaneous leishmaniasis, *HBV/HCV* hepatitis B/C, *HIV* Human immunodeficiency virus

### Immunopathogenesis paradigms for CL

For decades, the classic Th1/Th2 and M1/M2 paradigms have been applied to immune responses to *Leishmania* infections including the diverse clinical presentations of CL [19, 20]. Among these, the Th1-based model describes self-healing localized lesions to be the result of a strong protective Th1 response, balanced by regulatory mechanisms (notably IL-10), that successfully eliminates the parasite whilst minimizing pathology [4]. At one polar extreme of this proposed spectrum are the difficult to treat nodular lesions in ‘DCL’ patients which are characterized by a low to absent Th1 response resulting in a high parasite load. At the other polar extreme are mucosal lesions, as seen in ‘ML’ and ‘MCL’ patients, that result from an exacerbated inflammation with high numbers of cytotoxic CD8^+^ T cells. Generally, humoral (B cells and antibodies) and T cell-mediated (Th1, delayed-type hypersensitivity and cytotoxicity) immune responses are described as inversely related, whereas IL-10 production and parasite load in lesions are positively correlated. Several challenges emerge when implementing this model in the Ethiopian context. As mentioned earlier, one major issue is that the restricted classifications do not adequately capture the clinical spectrum of Ethiopian CL, complicating and biasing direct comparisons. In addition, this widely cited Th1-based paradigm rests largely on mouse models for a different set of *Leishmania* species (*L. major, L. braziliensis, L. amazonensis,* and *L. mexicana*), excluding *L. aethiopica* which is the main parasite species to cause CL in Ethiopia [4].

Nevertheless, underlying immune responses may still contribute to the diverse clinical spectrum of Ethiopian CL. However, so far, research into both humoral and T cell-mediated responses in Ethiopian CL has been relatively scarce and often lack comprehensive detail. For instance, regarding the humoral response, while plasma cells were observed in lesions they were never quantified, not allowing comparison between CL patient groups [6, 7, 21–23]. The few available studies on circulating *Leishmania*-specific antibodies also found no significant differences between ‘LCL’ and ‘DCL’ patients versus endemic healthy individuals [7, 22]. While relatively more studies have examined T cell-mediated responses in Ethiopian CL, they have largely focused on assessing the proliferation and the pro- and anti-inflammatory cytokine expression of circulating immune cells with and/or without *Leishmania* (extract) stimulation [15, 24–29]. The compilation of these studies have also not shown a clear correlation with the Th1-based paradigm underlying disease progression. Conflicting results between Ethiopian CL studies including non-Good Manufacturing Practice (non-GMP) manufactured *Leishmania* extracts [Soluble *Leishmania* Antigen (SLA)] may stem from variability in used extracts (e.g., differences between promastigote and amastigote cultures or diversity in *Leishmania* strains). Such *Leishmania* extracts, also known as Leishmanin, have historically been used to elicit a delayed-type hypersensitivity response (the ‘Leishmanin skin test’ or LST), reflecting the activation and expansion of skin tissue resident memory T cells [6, 7, 22]. However, these studies observed no difference between ‘LCL’ and ‘DCL’ patients. To date, the role of other immune cells such as monocytes/macrophages, natural killer cells and neutrophils have been limitedly investigated in Ethiopian CL, and available studies primarily focus on circulating rather than lesional cell subsets [27, 30].

While studies on circulating immune cells are valuable for identifying infiltrating immune cells and assessing the potential systemic spread from a localized skin infection, to gain insights into the underlying immunopathogenesis of Ethiopian CL, it is essential to study the immune mechanisms within the lesion. To date, few immunological studies on Ethiopian CL lesions have been performed, with only Nilsen and Hana in 1987 providing a semi-quantitative analysis of immune cell types using immunohistochemistry [6, 23, 31]. By quantifying and characterizing the immune response within lesions, future clinico-immunological studies can determine presence of consistent ‘immune patterns’ (immunotypes) corresponding to certain clinical presentations (Table 1), and further verify whether these insights can be employed to develop more suitable classifications and treatment strategies for Ethiopian CL.

### Parasite diversity

Since the clinical presentations and host immune responses may be *Leishmania* species-specific, parasite species identification should be integrated in future clinico-immunological studies. Historically, Ethiopian CL was ascribed to an infection with *L. aethiopica* without confirmed species identification, a reflection of limited diagnostic resources. While most infections are still likely due to *L. aethiopica,* its exclusive role has been increasingly questioned, as recent reports document other *Leishmania* species causing CL in Ethiopia [32]. Over the past decades, sporadic reports of CL caused by *L. major, L. infantum, L. donovani* and *L. tropica* have been repetitively mentioned in manuscripts though many lack clear references. While *L. major* was reported as early as 1973, only a single study has identified this species in *Phlebotomus duboscqi* in Southern Ethiopia, with no confirmed human infections to date [33, 34]. *L. infantum* and *L. donovani* are commonly known to cause visceral leishmaniasis and the associated post-kala-azar dermal leishmaniasis. However, in Ethiopia, there is only one report of *L. infantum* in dogs with no human infections mentioned to date [32]. Meanwhile, first human CL cases due to *L. donovani* have emerged in recent years [35]. CL caused by *L. tropica* was first documented in 2006 and recently identified in an outbreak among militia in Ethiopia’s Somali region [36, 37]. These sporadic reports offer limited clinical descriptions and lack immunological details, making the ability to establish associations between clinical presentations, underlying immune responses and infecting parasite species difficult. Further contributing to this parasite diversity, the *L. aethiopica* species exhibits a significant genetic variability, including evidence of hybridization with other *Leishmania* species (e.g., *L. donovani* and *L. tropica*)[38, 39]. However, using whole genome sequencing, a recent study found no associations between certain clinical classifications and phylogenetic clusters in *L. aethiopica* isolates [15]. Another group detected *Leishmania* RNA virus (LRV) in half of the investigated *L. aethiopica* isolates [40]. Presence of LRV has been associated with disease severity in other *Leishmania* species [41], but comparisons between clinical presentations, host immune response and LRV infection are still lacking within the Ethiopian context.

Considering the diverse range of clinical presentations and how certain immunopathogenic mechanisms may be strain-specific, parasite species identification, and even further characterization, should be a standard component for future clinico-immunological studies in Ethiopian CL. A variety of techniques are available for species identification, each with varying sensitivities, including isoenzyme electrophoresis, polymerase chain reaction (PCR)-restriction fragment length polymorphism (RFLP), and internal transcribed spacer 1 (ITS-1 PCR) with high-resolution melt (HRM) analysis [2, 35]. Further in-depth analysis to assess LRV (e.g., quantitative reverse transcription-PCR) and *Leishmania* phylogenetic clusters (e.g., whole genome or transcriptome sequencing) can aid in identifying confounding factors [39, 42, 43]. Lastly, while characterizing parasite isolates from cultures can provide a higher yield, it may not accurately represent the in vivo parasite population, as minority (slower-growing) strains could be inadvertently selected against.

## Key considerations to efficiently address knowledge gaps

### Multidimensional approach

To provide better insights of Ethiopian CL, future studies are recommended to include a multidimensional approach comprising thorough clinical, immunological, and parasitological assessments. Clinical data collection forms, as proposed in Table 1, allow consistent and in-depth data documentation across cohorts. Clinical study protocols employing such data collection can be registered in public platforms such as ClinicalTrials.gov or Pan African Clinical Trials Registry (PACTR) or disseminated through peer-reviewed publications, allowing others to align and standardize their clinical studies [11, 17]. From a sampling perspective, lesion-derived material should be prioritized for both immunological profiling and parasite characterization, as it offers a direct insight at the source of infection. To assess in-patient heterogeneity, sampling multiple lesions can be considered. When sampling punch biopsies from larger lesions, the exact sampling site should be documented, as spatial variation (e.g., mucosal versus non-mucosal areas, central versus peripheral lesional regions) may influence immune cell composition and parasite load. Blood samples can still provide complementary information on potential systemic immune responses. Ethical approvals have already been obtained for clinical studies on Ethiopian CL including such sampling strategies [11, 17]. By collecting standardized clinical data and sufficient representative biological samples from the same individuals, inter-study variability will be significantly reduced. Both host- and parasite-related confounders can be accounted for, allowing for appropriate cross-cohort comparisons.

### Public databases and biobank inventory

Appropriate comparisons across cohorts from different studies on Ethiopian CL can also be achieved through verifying findings using either publicly available datasets, or archived clinical samples remaining from prior studies, provided that patient consent was obtained via informed consent forms for secondary analyses. Given the heterogeneity in how clinical presentations of CL are assessed and classified by different physicians, we strongly recommend sharing the elaborate clinical database, as well as anonymized lesion photographs to detail used classifications. To facilitate such downstream data sharing, for future clinical studies, it is recommended to implement a granular consent model at the informed consent form stage, including separate sub-consent options for secondary use of clinical data and of anonymized lesion images. Data access procedures, typically managed through the institution’s Data Access Committee and formalized via a Data Sharing Agreement, should be made transparent via the journal’s open-science platform or by linking them on the study’s ClinicalTrials.gov registration, with appropriate measures taken to safeguard patient privacy, especially for image-based data.

Anonymization of lesion images should prioritize cropping (Fig. 1) or blurring non-lesional areas [12], rather than masking the eyes with a black bar, and all image metadata (e.g., embedded geolocation data) should be permanently removed before storage or sharing. Image ownership and reuse should follow the journal and/or institutional licensing policy (e.g. Creative Commons licenses permitting reuse with correct referencing). Properly archived and consented lesion image datasets can subsequently support artificial intelligence-assisted pipelines for automated lesion characterization [44], enabling the development of more objective and reproducible clinical classification systems for Ethiopian CL while reducing annotation workload and observer bias.

Moreover, raw sequencing data, whether from the parasite (e.g., whole genome sequencing) or the host (e.g., transcriptomic signatures), should be deposited in publicly accessible repositories such as the European Nucleotide Archive (ENA), the Sequence Read Archive (SRA), or Gene Expression Omnibus (GEO). This allows for downstream analysis when updated bioinformatics tools, reference genomes or annotation datasets become available, thereby maximizing the long-term utility and reproducibility of the data.

Continuous methodologic advancements enable high-resolution analyses of archived Ethiopian CL samples, maximizing biobank potential without additional patient burden. For instance, lesional scrapings and their extracted DNA, if stored properly and in adequate quantity, can still be used for molecular analyses, allowing for more in-depth sequencing at a later time. Similarly, lesion biopsies stored in formalin-fixed paraffin-embedded (FFPE) blocks or snap-frozen in liquid nitrogen can be preserved for years, and thus remain valuable resources for subsequent histopathological and transcriptomic analyses. A centralized, open-access platform referencing available sample biobanks linked to published studies would facilitate this by allowing researchers to request tailored analyses of archived samples, similar to what is currently in place for lung samples by the Tuberculosis Research Resource from the University of Alabama at Birmingham. Other existing initiatives such as the Infectious Diseases Data Observatory (IDDO), which promotes equitable data sharing for neglected and emerging infectious diseases, and the Foundation for Innovative New Diagnostics (FIND), which supports biobanking for diagnostic testing and product development, provide valuable frameworks and potential partners for advancing open access to data and clinical samples of past Ethiopian CL studies.

### Local investments

Over the past decade, there have been substantial improvements in the Ethiopian research infrastructure, largely driven by international funding mechanisms (e.g., the World Bank) and large research projects [e.g., European and Developing Countries Clinical Trials Partnership (EDCTP), NIH]. Global North–South collaborations, including studies focused on Ethiopian leishmaniasis research, both visceral leishmaniasis and CL, have invested in crucial laboratory equipment ranging from biosafety cabinets to advanced flow cytometers, which are often donated to host institutions after project completion [45, 46]. Other initiatives, such as the ‘Structured Operational Research and Training Initiative’ (SORT IT) coordinated by ‘WHO Special Programme for Research and Training in Tropical Diseases’ (TDR WHO) and implemented with global partners, have made substantial contributions to strengthening local research capacity by training health professionals to conduct operational research on neglected tropical diseases in Ethiopia [47]. While the practical training of Ethiopian researchers significantly advance local research capabilities, major ongoing challenges of these local investments are the emigration of highly trained academic personnel, and the continued maintenance of donated equipment. To mitigate this, one should strongly focus on training and retaining skilled clinical and technical staff who can apply and transfer their expertise to future local studies (‘training the trainers’). For instance, in 2004, the *Leishmania* Research and Treatment Center (LRTC, University of Gondar, Ethiopia) was established by the Drugs for Neglected Diseases Initiative (DNDi) and has trained a dedicated team of clinical and technical staff, many of whom have remained over the years. Building on this success, the University of Gondar, DNDi and the Institute of Tropical Medicine Antwerp (Belgium) established the LRTC-affiliated Clinical Trial and advanced Research Laboratory, expanding local research capacity to Good Clinical Practice (GCP) and Good Clinical Laboratory Practice (GCLP)-compliant standards. Moreover, a national clinical network of Ethiopia (CTN-ET) comprising of multiple clinical trial units across the country has recently been established to further promote, advocate and coordinate these GCP and GCLP-compliant clinical trials across the nation. Such research facilities are essential to preserve and manage the donated equipment, maximizing its usability and longevity. Complementing this, a clinical research network is currently harmonizing guidelines on leishmaniasis diagnosis and treatment, by updating the existing guidelines published in 2013 [9], which will eventually inform and update (inter)national reports on Ethiopian CL.

## Conclusions

Investigating the immune responses underlying Ethiopian CL is essential to unravel its diverse disease mechanisms, and represents a priority in the WHO strategies for controlling CL. However, long-standing assumptions in the research field have shaped biased views of the potential immune mechanisms driving different clinical presentations, which remain unverified in the Ethiopian context. In this opinion, we outline key considerations, including the importance of standardized and extensive clinical documentation, and the strategic collection of clinical samples tailored for both in-depth immunological and parasitological analyses. Equally important is the integration of capacity strengthening initiatives, particularly when employing advanced, high-throughput technologies, to ensure that research expertise is not only shared but also locally retained for future studies. The recent establishment of national networks represents a critical step toward harmonizing guidelines and maintaining GCP and GCLP-standards across Ethiopia. Promoting data sharing and equitable access to archived samples, alongside capacity strengthening efforts, will facilitate appropriate cross-study comparisons and enable more in-depth downstream analyses. By reducing clinical assessment and sampling bias, improving cross-cohort comparisons, strengthening in-country analytical capacity while supporting long-term retention of trained personnel, these recommendations help establish a more comprehensive, unbiased and sustainable research environment.

## Data Availability

In line with the journal’s Creative Commons Attribution 4.0 International (CC BY 4.0) license, the lesion images included in this article may be shared and reused with appropriate attribution. Access to the original lesion images is available from the corresponding author on reasonable request. The complete clinical dataset together with de-identified lesion pictures of the Spatial CL study can be requested via the Data Access Committee of the Institute of Tropical Medicine Antwerp (https://www.itg.be/en/research/data-sharing-and-open-access) via a formal application and signed Data Sharing Agreement. In line with the study’s data sharing policy, these datasets will be made available one year after completion of all planned primary publications, currently projected for 2028.

## Abbreviations

CL: Cutaneous leishmaniasis
CTRL: Clinical Trial and advanced Research Laboratory
CTN-ET: Clinical Trials Network–Ethiopia
DCL: Diffuse cutaneous leishmaniasis
EDCTP: European and Developing Countries Clinical Trials Partnership
FIND: Foundation for Innovative New Diagnostics
GCP: Good Clinical Practice
GCLP: Good Clinical Laboratory Practice
HRM: High-resolution melt analysis
IDDO: Infectious Diseases Data Observatory
ITS-1: Internal transcribed spacer 1
LCL: Localized cutaneous leishmaniasis
LRV: *Leishmania* RNA virus
LST: Leishmanin skin test/Montenegro skin test
M1: Classically activated (pro-inflammatory) macrophage phenotype
M2: Alternatively activated (tissue-repair) macrophage phenotype
MCL: Mucocutaneous leishmaniasis
ML: Mucosal leishmaniasis
NIH: National Institutes of Health (US)
PCR: Polymerase chain reaction
RFLP: Restriction fragment length polymorphism
SORT IT: Structured Operational Research and Training Initiative
TDR WHO: WHO Special Programme for Research and Training in Tropical Diseases
Th1: T helper 1 response
Th2: T helper 2 response
